# Environmental Variables Shaping the Ecological Niche of *Thaumarchaeota* in Soil: Direct and Indirect Causal Effects

**DOI:** 10.1371/journal.pone.0133763

**Published:** 2015-08-04

**Authors:** Jin-Kyung Hong, Jae-Chang Cho

**Affiliations:** Institute of Environmental Sciences and Department of Environmental Sciences, Hankuk University of Foreign Studies, Yong-In, Korea; CAS, CHINA

## Abstract

To find environmental variables (EVs) shaping the ecological niche of the archaeal phylum *Thaumarchaeota* in terrestrial environments, we determined the abundance of *Thaumarchaeota* in various soil samples using real-time PCR targeting thaumarchaeotal 16S rRNA gene sequences. We employed our previously developed primer, THAUM-494, which had greater coverage for *Thaumarchaeota* and lower tolerance to nonthaumarchaeotal taxa than previous *Thaumarchaeota*-directed primers. The relative abundance estimates (RVs) of *Thaumarchaeota* (R_THAUM_), *Archaea* (R_ARCH_), and *Bacteria* (R_BACT_) were subjected to a series of statistical analyses. Redundancy analysis (RDA) showed a significant (*p* < 0.05) canonical relationship between RVs and EVs. Negative causal relationships between R_THAUM_ and nutrient level–related EVs were observed in an RDA biplot. These negative relationships were further confirmed by correlation and regression analyses. Total nitrogen content (TN) appeared to be the EV that affected R_THAUM_ most strongly, and total carbon content (TC), which reflected the content of organic matter (OM), appeared to be the EV that affected it least. However, in the path analysis, a path model indicated that TN might be a mediator EV that could be controlled directly by the OM. Additionally, another path model implied that water content (WC) might also indirectly affect R_THAUM_ by controlling ammonium nitrogen (NH_4_^+^-N) level through ammonification. Thus, although most directly affected by NH_4_^+^-N, R_THAUM_ could be ultimately determined by OM content, suggesting that *Thaumarchaeota* could prefer low-OM or low-WC conditions, because either of these EVs could subsequently result in low levels of NH_4_^+^-N in soil.

## Introduction

The discovery of ammonia-oxidizing archaea (AOA) has changed a century-old paradigm whereby the first step in the nitrification process, chemolithotrophic ammonia oxidation, was thought to be performed exclusively by ammonia-oxidizing bacteria (AOB) belonging to *β-* and *γ-Proteobacteria*. Archaeal homologs to the bacterial ammonia monooxygenase gene (*amoA*) were found in environmental genomes, providing initial evidence indicating an archaeal contribution to ammonia oxidation [1, 2], and at the same time, a marine AOA strain, *Nitrosopumilus maritimus* SCM1, was first isolated from a saltwater aquarium by enrichment and filtration techniques [3]. AOA were initially classified as mesophilic *Crenarchaeota* [1, 2], but in 2008, the third archaeal phylum, *Thaumarchaeota*, was proposed, based upon archaeal phylogeny inferred from rRNA and ribosomal protein sequences, to distinguish the mesophilic AOA lineages from hyperthermophilic *Crenarchaeota* lineages [4]. Few years later, this distinction was confirmed by genomic information (e.g., the identification of *Thaumarchaeota*-specific genes) in *Cenarchaeum symbiosum*, *Nitrosopumilus maritimus*, and *Nitrososphaera gargensis*, representatives of marine and terrestrial AOA lineages [5]. To date, ten thaumarchaeotal species (*Cenarchaeum symbiosum*, *Nitrosoarchaeum koreensis*, *Nitrosoarchaeum limnia*, *Nitrosocaldus yellowstonii*, *Nitrosopumilus maritimus*, *Nitrosopumilus salaria*, *Nitrososphaera gargensis*, *Nitrososphaera viennensis*, *Nitrosotalea devanaterra*, and *Nitrosotenuis uzonensis*) have been isolated from marine and terrestrial environments, and have been well characterized [3, 6–16]. However, although extensive effort has been devoted to understanding the biochemical, physiological, and genomic characteristics of *Thaumarchaeota*, our knowledge of the ecology of *Thaumarchaeota* is relatively limited. Although findings from ecological studies so far conducted upon *Thaumarchaeota* have been well discussed in conjunction with its physiology in several review articles [17–19], the ecological niche of *Thaumarchaeota* remains largely unknown. It is important to know the lifestyle of *Thaumarchaeota* in environments to fully understand this phylum, as well as to provide basal information facilitating further exploration of its as yet unrecognized aspects.

Clues required to elucidate the ecological niche of *Thaumarchaeota* have been obtained mainly from relative abundance estimates of *Thaumarchaeota* based on the ratio of the copy number of archaeal *amoA* to that of bacterial *amoA* (i.e., the *amoA*_ARCH_/*amoA*_BACT_ ratio) under the assumption that all archaeal *amoA*-carrying prokaryotes are *Thaumarchaeota* and vice versa [20–27]. However, the conclusions of studies based upon the use of this ratio might have two limitations. First, although it is useful to differentiate the niche of AOA from the niche of AOB, the *amoA*_ARCH_/*amoA*_BACT_ ratio cannot provide substantive information on the *Thaumarchaeota*-specific niche. The *amoA*_ARCH_/*amoA*_BACT_ ratio reflects only the response of AOA to EVs relative to the analogous response of AOB, and not relative to that of all other nonthaumarchaeotal taxa. Second, the PCR primer pairs currently used to target archaeal *amoA* may have limited coverage. A high level of sequence variations (identities < 85%) in *amoA* has been reported [28], and specificity problems of *amoA*-targeting primers have been pointed out [29, 30]. In addition to these main limitations, the phylum *Thaumarchaeota* might contain members that are deficient in ammonia oxidation activity [31]. Since almost all methods currently used for cultivating the members of *Thaumarchaeota* use ammonia as a sole energy source, the members thus cultured could include only ammonia-oxidizing strains. Similarly, as yet undiscovered archaeal groups other than *Thaumarchaeota* might possess archaeal *amoA* xenologs.

An alternative approach to determine the *Thaumarchaeota*-specific ecological niche is to use a quantitative method targeting a universally applicable housekeeping gene such as the 16S rRNA gene, the copy number of which can provide a good abundance estimate regardless of the particular metabolic characteristics of the organisms under study. However, when using this 16S rRNA gene–based approach, it could be difficult to obtain information particularly regarding the response of *Thaumarchaeota* (AOA) to EVs compared to that of only AOB, unless the abundance data for AOB were collectively obtained from different AOB phylogenetic groups (e.g., β-proteobacterial AOB and γ-proteobacterial AOB). Nonetheless, the 16S rRNA gene–based approach provides very important information especially regarding the response of *Thaumarchaeota* to EVs compared to that of all other prokaryotic groups, facilitating identification of the *Thaumarchaeota*-specific niche. However, in our previous study [32], 16S rRNA gene–directed PCR primer pairs frequently used for quantifying *Thaumarchaeota*, 771F-957R and MCGI391F-MCGC554R (identical to primer pair MGI391-Cren537) [33, 34], showed unsatisfactory coverage for *Thaumarchaeota*. These primer pairs captured sequences belonging to only a single thaumarchaeotal subgroup (either marine group I [MG-I] or soil crenarchaeotic group [SCG]), with insufficient coverage (MCGI391F-MCGC554R, 47.5% of MG-I sequences; 771F-957R, 87.3% of SCG sequences). All previous 16S rRNA gene–based ecological studies upon *Thaumarchaeota*, except for those studies using a next-generation sequencing approach that employed universal primers, implicitly assumed that MG-I and SCG predominated in marine and terrestrial environments, respectively, and included the use of a primer pair specific to one of these thaumarchaeotal subgroups for estimating the abundance of *Thaumarchaeota* (or AOA). However, unexpected thaumarchaeotal subgroups (e.g., subgroups MG-I in the terrestrial environment and subgroup SCG in the marine environment) might reside in the environment under study, as observed by Tourna *et al*. [30] and Beman and Francis [35]. Moreover, multiple subgroups or even as yet undiscovered subgroups might possibly reside together. In samples obtained from such environments, the abundance of *Thaumarchaeota* could be underestimated. In addition, the thaumarchaeotal abundance measured by using the primer pair 771F-957R could be considerably overestimated due to this primer pair’s high tolerance (the extent to which it binds to nontarget taxa, ~36.4%) [32].

In this study, to determine EVs shaping the ecological niche of *Thaumarchaeota*, we estimated its abundance in various soil samples by using real-time PCR that targeted 16S rRNA gene sequences. We employed our newly developed PCR primer, THAUM-494, which has greater coverage (92.9%) for *Thaumarchaeota* and lower tolerance (0.9%) to nonthaumarchaeotal taxa than other primers targeting this phylum, as we have reported previously [32]. The abundance estimate of *Thaumarchaeota* was compared with that of total prokaryotes, and was subjected to a series of statistical analyses. The EVs affecting the relative abundance of *Thaumarchaeota* in soil were identified, and their direct and indirect causal effects are described in this paper.

## Materials and Methods

### Soil sampling and DNA extraction

Twenty-seven soil samples were collected from sites with and without vegetation (grassland, 25.9%; forest, 40.7%; arid soil, 25.9%) in Korea. Soil samples belonged to andisol (11.1%), entisol (51.9%), inceptisol (14.8%), and ultisol (18.5%), and the majority (70.4%) of them were sandy loam. The soil samples were taken below 10 cm from the soil surface, after surface litter (e.g., plant debris) was removed. All sampling sites were located in private land. We obtained landowners’ permission to collect soil samples from their property land prior to sampling.

After sieving (sieve size = 2 mm) on site, the soil samples were immediately stored at 4°C for transportation to the laboratory, and were subjected to DNA extraction upon arrival. Community DNAs were directly extracted from the soil samples by using a PowerSoil DNA isolation kit (MoBio Laboratories, Carlsbad, CA, USA) according to the manufacturer’s protocol. To avoid subsample bias, DNA obtained from four subsamples per soil sample was pooled to prepare each sample’s PCR template.

### Physicochemical analysis

Soil temperature was measured at ca. 10 cm depth by using a bimetallic thermometer. Water content (WC) was determined from the percent weight loss after oven-drying at 105°C for 18 h. Soil pH was determined as pH_1:5_ after mixing with distilled water (soil-to-water ratio, 1:5 [w/v]). Total carbon (TC) content was measured by a combustion method [36] by using a carbon analyzer (TOC-L, Shimadzu, Kyoto, Japan) equipped with a combustion module (SSM5000A, Shimadzu). Total nitrogen (TN) content was determined by means of Kjeldahl digestion [37]. Total phosphorus (TP) and total sulfur (TS) contents were determined by using an inductively coupled plasma–atomic emission spectrometer (ICP-AES 7510, Shimadzu), after extraction of samples using a hydrogen chloride and nitric acid solution [38–41]. Ammonium nitrogen (NH_4_^+^-N) and nitrate nitrogen (NO_3_^−^-N) were respectively determined by means of Kjeldahl digestion [36] and ion chromatography (ICS 5000, Thermo Fisher Scientific, Sunnyvale, CA, USA) [42, 43].

### Real-time PCR

Abundance estimates of *Thaumarchaeota* were determined by using a real-time PCR (RTi-PCR) assay employing the *Thaumarchaeota*-specific primer THAUM-494, which we developed previously, and demonstrated to be more inclusive and specific than other primers used for quantifying *Thaumarchaeota*, in terms of coverage for *Thaumarchaeota* and tolerance to nonthaumarchaeotal taxa [32]. THAUM-494 was paired with an archaeal universal primer 917R [44]. The primer pair (THAUM-494-917R) used in this study had a higher coverage (89.3%) for *Thaumarchaeota* and lower tolerance (0.9%) to nonthaumarchaeotal taxa than previously used primer pairs (coverage, 21.9–33.7%; tolerance, ~36.4%) (S1 Table); thus our primer pair was expected to provide a considerably better estimate of the abundance of *Thaumarchaeota*. Primer pairs ARC806–ARC915 [45, 46] and Eub338–BAC515 [47, 48] were used for quantifying *Bacteria* and *Archaea*, respectively (S2 Table lists coverage values of the universal primers used).

SYBR Premix Ex Taq reagent (Takara, Shiga, Japan) was used for the RTi-PCR. SYBR Green I and ROX were used as reporter and passive reference dyes, respectively. Reactions were carried out in MicroAmp optical eight-tube strips (Applied Biosystems, Foster City, CA, USA) by using a ABI Prism 7300 sequence detection system (Applied Biosystems). Each reaction contained 2 μl of template DNA, 25 μl of SYBR Premix Ex Taq, 1 μl of ROX dye, 1 μl (20 pmol) of each primer, and sterile water to bring the total reaction volume to 50 μl. Thermal cycling parameters were as follows: initial denaturation (95°C) for 5 min, followed by 35 cycles of denaturation (95°C) for 30 s, primer annealing (primer pairs for *Thaumarchaeota*, 55°C; *Archaea*, 60°C; *Bacteria*, 55°C) for 30 s, and final extension (72°C) for 30 s. Fluorescence signals were measured during the extension step. RTi-PCR amplifications were performed in triplicate. The collected fluorescence signals were analyzed by using ABI Sequence Detection Software version 1.4 (Applied Biosystems). The fluorescence signal intensity of the reporter dye was normalized by using the signal intensity of the passive reference dye to correct for fluctuations in the fluorescence signal due to the changes in the concentration and volume of the reaction mixture. The threshold cycle (C_T_) was taken to be the PCR cycle at which a significant increase in the normalized fluorescence signal was first detected. The threshold level was determined automatically by the detection system, using the default settings.

Recombinant plasmid DNAs were used to construct standard curves for determining the copy numbers of 16S rRNA gene sequences of *Thaumarchaeota*, *Archaea*, and *Bacteria* (S1 Fig). Purified DNAs from environmental clones obtained in our preliminary study, which contained 16S rRNA gene sequences of *Thaumarchaeota*, *Archaea*, and *Bacteria* (GenBank accession numbers KF275705, KF276604, and GQ143752, respectively) were used as template DNAs for constructing the standard curves. RTi-PCR amplifications used in constructing the standard curves were performed in quadruplicate using a range of template DNA concentrations (ca. 10^1^–10^9^ copies of 16S rRNA gene sequences per reaction). The slopes of the standard curves ranged from −3.29 to −3.20 (R^2^ = 0.994–0.999), indicating PCR efficiency near 100% (PCR efficiency = e^−ln(10)/slope^ − 1).

### Statistical Analysis

Based on the copy numbers of 16S rRNA genes of *Thaumarchaeota* (N_THAUM_), *Archaea* (N_ARCH_), and *Bacteria* (N_BACT_) determined in RTi-PCR assays, relative abundance estimates of *Thaumarchaeota* (R_THAUM_), *Archaea* (R_ARCH_), *Bacteria* (R_BACT_) were calculated as R_THAUM_ = N_THAUM_/N_PROK_, R_ARCH_ = N_ARCH_/N_PROK_, and R_BACT_ = N_BACT_/N_PROK_, respectively, where N_PROK_ = N_ARCH_ + N_BACT_. Multivariate statistical analyses were performed to establish relationships between the relative abundance estimates (RVs) and environmental variables (EVs). Log-transformed data (except pH data) were subjected to RDA [49] using RVs as response variables and EVs as explanatory variables, in which the ordination of RVs is constrained in a way such that the resulting ordination vectors are linear combinations of the EVs [50]. For statistical ordination, we preferred RDA employing a linear model rather than canonical correspondence analysis employing a unimodal model [51] because the maximum length of the gradient determined by means of detrended correspondence analysis [52] was less than two standard deviations [53]. The statistical significance of the overall RDA results was assessed (α = 0.05) by a permutation test (of 499 iterations) on the null hypothesis that the RVs and EVs are not linearly related. In a resulting RDA biplot, the lengthening factor of 1.5 was applied to EV vectors to improve the legibility of the biplot. Pearson correlation coefficients (*r*) between the RVs and EVs were calculated to quantify the symmetric relationship between them, and the dependency (asymmetric relationship) between them was individually evaluated by means of simple linear repression analyses using the ordinary least squares (OLS) method. Multiple linear regression (MLR) analysis using the OLS method was also performed where appropriate.

To determine the direct and indirect effects of EVs upon R_THAUM_, path analysis [54, 55] was performed. In a path model, path coefficients (PCs) were calculated using OLS-MLR to assess the contributions of EVs to R_THAUM_ by means of both direct and indirect paths. The significance of the path model was evaluated by using the goodness of fit chi-square (χ^2^_GoF_) as well as the root mean square error of approximation (RMSEA). In each significant path model, covariations among variables were decomposed to distinguish noncausal covariation from total covariation. All statistical data analyses were performed using the software packages PAST (Paleontological Statistics, Natural History Museum, University of Oslo, Oslo, Norway), Ωnyx (University of Virginia and Max Planck Institute for Human Development, http://onyx.brandmaier.de), IBM SPSS-AMOS (IBM Software, Armonk, NY, USA), and XLSTAT (Addinsoft, Paris, France). All software packages were used according to the instructions in their manufacturers’ manuals.

## Results and Discussion

### Physicochemical and microbiological variables

In total, 27 soil samples were collected from various terrestrial sites; Table 1 summarizes their physicochemical properties. In our samples, variations of the EVs studied were measured as relative standard deviations (RSDs), and ranged from 10.5% to 196.1% (average 102.4%). Soil pH varied the least among samples, and the largest RSD was observed for TN. In every soil sample analyzed, the total inorganic carbon content was below the detection limit; thus we considered the total organic carbon content (TOC) to be equal to the TC; the TOC in turn was taken to reflect the OM content. The correlation coefficient between TC and OM was 0.814 (*p* < 0.05) in our preliminary study.

**Table 1:** Summary of physicochemical and microbiological properties of soils used in this study.

			Average	Standard deviation	Median	Maximum	Minimum	IQR^a^	RSD^b^
Physicochemical variables^c^	Temperature (Temp)		22.7	7.2	22.0	37.0	6.0	7.5	3.2 × 10^−1^
	pH		6.1	6.4 × 10^−1^	6.4	7.0	4.7	6.3 × 10^−1^	1.1 × 10^−1^
	Water content (WC)		22.1	12.7	18.6	56.1	8.5	11.4	5.8 × 10^−1^
	Total carbon (TC)		9.8	12.6	5.6	49.3	8.7 × 10^−1^	6.0	1.3
	Total nitrogen (TN)		2.5	4.8	6.8 × 10^−1^	20.1	1.5 × 10^−1^	1.0	2.0
		Ammonium-nitrogen (NH_4_^+^-N)	0.2	0.4	7.2 × 10^−2^	1.7	2.1 × 10^−2^	8.1 × 10^−2^	1.9
		Nitrate-nitrogen (NO_3_^−^-N)	1.5 × 10^−1^	1.4 × 10^−1^	9.6 × 10^−2^	5.2 × 10^−1^	4.0 × 10^−2^	4.2 × 10^−2^	9.5 × 10^−1^
	Total phosphorus (TP)		8.9 × 10^−1^	5.9 × 10^−1^	6.9 × 10^−1^	2.0	5.8 × 10^−2^	9.6 × 10^−1^	6.6 × 10^−1^
	Total sulfur (TS)		5.3 × 10^−1^	7.6 × 10^−1^	2.9 × 10^−1^	3.3	6.7 × 10^−2^	2.6 × 10^−1^	1.4
16S rRNA gene copy number^d^	*Thaumarchaeota* (N_THAUM_)		5.9 × 10^6^	5.8 × 10^6^	3.3 × 10^6^	1.8 × 10^7^	3.5 × 10^4^	8.1 × 10^6^	9.8 × 10^−1^
	*Archaea* (N_ARCH_)		1.1 × 10^7^	9.9 × 10^6^	8.6 × 10^6^	4.1 × 10^7^	7.0 × 10^4^	1.4 × 10^7^	8.7 × 10^−1^
	*Bacteria* (N_BACT_)		5.6 × 10^8^	5.9 × 10^8^	4.3 × 10^8^	2.8 × 10^9^	2.6 × 10^6^	6.9 × 10^8^	1.1
Relative abundance estimate^e^	*Thaumarchaeota* (R_THAUM_)		1.4	1.0	1.1	4.8	9.3 × 10^−2^	1.1	7.7 × 10^−1^
	*Archaea* (R_ARCH_)		2.7	1.5	2.4	6.3	7.3 × 10^−1^	1.7	5.5 × 10^−1^
	*Bacteria* (R_BACT_)		97.3	1.5	97.6	99.3	93.7	1.7	1.5 × 10^−2^

^a^ IQR, inter quartile range

^b^ RSD, relative standard deviation. RSD = SD/average, where SD = sample standard deviation.

^c^ Units of measurement: Temp, °C; WC, %; TC, TN, NH_4_^+^-N, NO_3_^−^-N, TP, and TS, mg/g dry soil.

^d^ Unit of measurement: 16S rRNA gene copy number/g dry soil.

^e^ R_THAUM_ = N_THAUM_/N_PROK_, R_ARCH_ = N_ARCH_/N_PROK_, and R_BACT_ = N_BACT_/N_PROK_, where N_PROK_ = N_ARCH_ + N_BACT_.

The abundances of *Thaumarchaeota* (N_THAUM_), *Archaea* (N_ARCH_), and *Bacteria* (N_BACT_) estimated based on their 16S rRNA gene copy numbers were 5.9 × 10^6^ ± 5.8 × 10^6^, 1.1 × 10^7^ ± 9.9 × 10^6^, and 5.6 × 10^8^ ± 5.9 × 10^8^ per gram of dry soil, respectively. N_THAUM_ ranged from 3.5 × 10^4^ to 1.8 × 10^7^, and had 97.6% RSD. On average, *Archaea* constituted 2.7% of total prokaryotes in our soil samples, and 58.2% of *Archaea* (N_THAUM_/N_ARCH_) were estimated to be *Thaumarchaeota*. The proportion of *Thaumarchaeota* among total prokaryotes in the soil samples (R_THAUM_) ranged from 0.09 to 4.8% (average 1.4%). The RSD of R_THAUM_ was similar to that of R_ARCH_, but much larger (51×) than that of the relative abundance estimate of *Bacteria* (R_BACT_).

Our results for the relative abundance of *Thaumarchaeota*, R_THAUM_, suggested that, in terms of abundance *per se*, *Thaumarchaeota* might not be a major constituent of soil prokaryotes in our soil samples, although it was a numerically dominant member of soil *Archaea*. Our results are consistent with those of Ochsenreiter *et al*. [34] and Lehtovirta *et al*. [56], who reported that *Thaumarchaeota* represented up to 5% of the total prokaryotes in many soils. However, although such abundance figures may seem small, evaluations of the contribution of *Thaumarchaeota* to soil ecosystems should include the consideration that all the currently recognized members of *Thaumarchaeota* (at least all cultured or enriched *Thaumarchaeota*) are considered to be ammonia oxidizers. Because of the low energy yields (ΔG^0′^ = −235 kJ/mol) produced in the oxidation of ammonia [57] relative to those of the complete oxidation of OM by organotrophs, ammonia oxidizers have to respire much more than organotrophs, resulting in a considerably larger contribution by *Thaumarchaeota* to geochemical cycling. Thus, in terms of their ecological importance, conversion factors might be applied to the number of *Thaumarchaeota* that are orders of magnitude higher than those for organotrophs. It should also be noted that *Thaumarchaeota* is the only prokaryotic phylum all of whose known members participate in the ammonia oxidation process, unlike *Proteobacteria*, only a few of whose members at the genus level are ammonia oxidizers (e.g., *Nitrosomonas*, *Nitrosospira*, *Nitrosolobus*, and *Nitrosococcus*).

### Multivariate causal relationship projected onto a reduced space

Multivariate causal relationships between the EVs and the RVs determined from our soil samples were inferred by means of a gradient analysis using RDA. Because the maximum length of gradient determined by using detrended correspondence analysis was less than 2 standard deviations, RDA was used in lieu of canonical correspondence analysis, which is more appropriate for data sets with maximum gradient lengths greater than 3–4 standard deviations and assumes an unimodal model for response variables. To avoid the multicollinearity problem (association between NH_4_^+^-N and TN) as well as to simplify our initial inference, the EVs of inorganic nitrogen contents (NH_4_^+^-N and NO_3_^−^-N) were not included in the RDA.

**Fig 1:**
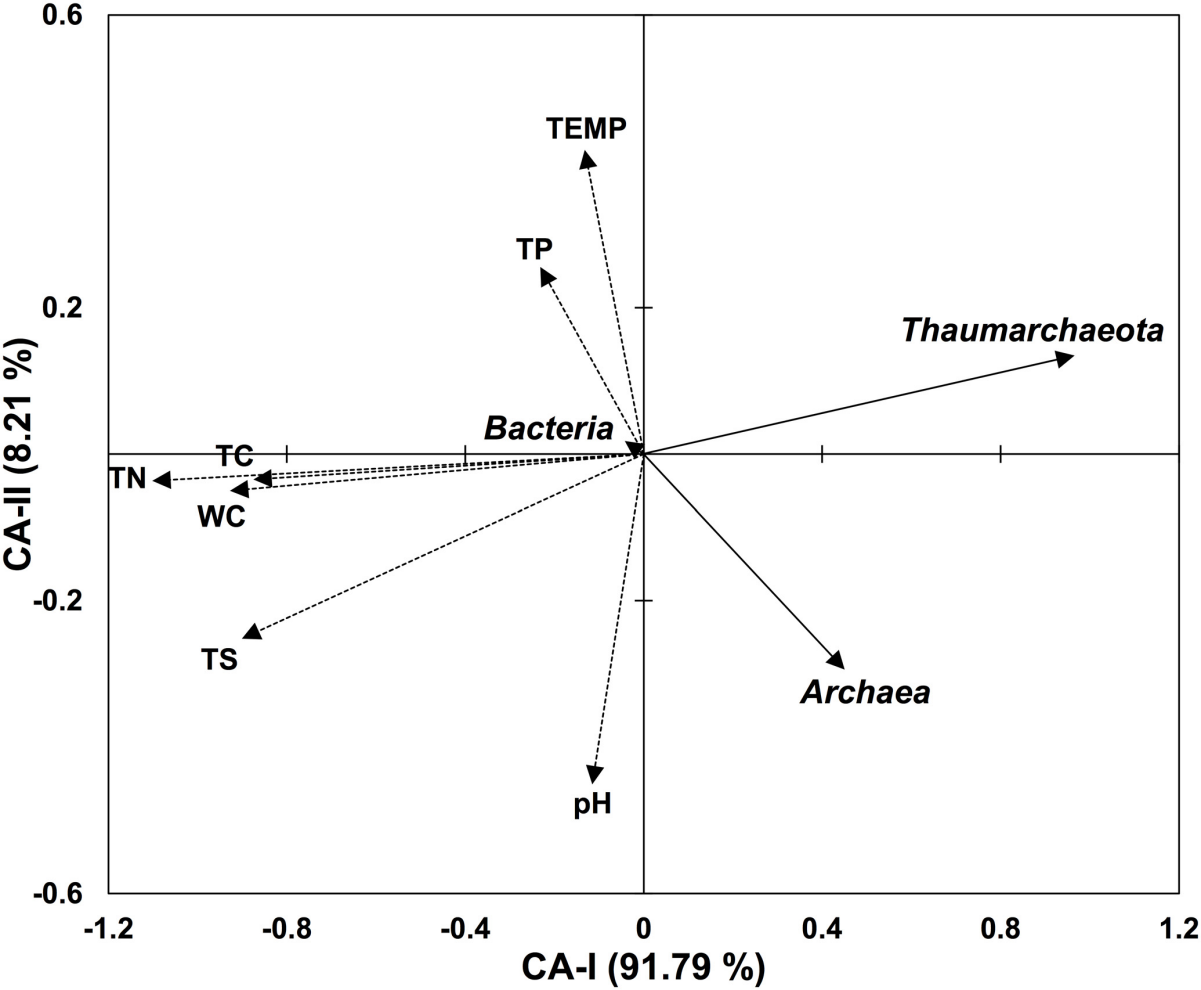
RDA biplot representing the relative abundance of prokaryotic taxa and environmental variables. Solid-line arrows and dashed-line arrows represent the biplot scores of the relative abundances of prokaryotic taxa and of the environmental variables, respectively. Values in parentheses indicate the percentages of the total variation that are explained by each canonical axis.

The test of significance on the overall RDA results performed using 499 permutations showed that the canonical relationship between the RVs and EVs was highly significant (*p* = 0.018), indicating that the relative abundance of prokaryotic taxa estimated in this study and the environmental variables measured in this study were linearly related. The two canonical axes (CA-I and CA-II) shown in the RDA biplot (Fig 1) explained almost all (>99.9%) the variance in the RV data set, and the first axis (CA-I) explained the great majority (91.5%) of the variance. The relative abundance of the phylum *Thaumarchaeota*, R_THAUM_, contributed the most (81.6%) to the first canonical axis, and the relative abundances of *Archaea* and *Bacteria* (R_ARCH_ and R_BACT_) respectively showed a moderate contribution (18.4%) and the smallest contribution (<0.001%) to the first canonical axis. The biplot score of R_BACT_ on the first canonical axis was very small (|−0.010|) compared to those of R_THAUM_ (|+0.957|) and R_ARCH_ (|+0.455|). Among the EVs, WC, TC, TN, and TS showed biplot scores greater than 0.9 on the first canonical axis, whereas temperature, pH, and TP showed biplot scores less than 0.3. In addition, when the causal effects of temperature, pH, and TP were controlled in partial RDA, the linear relationship between EVs and RVs was significant (*p* < 0.05), indicating that these EVs have marginal or no effect on RVs. The angles (directions) between RVs and EVs in the RDA biplot suggested negative causal relationships of R_THAUM_ with nutrition level–related environmental variables (TC, TN, and TS) and with WC. R_ARCH_ was also shown to be negatively related to these four EVs (TC, TN, TS, and WC), but the causal relationships between R_ARCH_ and these EVs appeared to be less strong than the relationships between the R_THAUM_ and these EVs.

We considered that raw values of thaumarchaeotal abundance estimates (N_THAUM_, thaumarchaeotal 16S rRNA gene copy number) could be a function of EV effects specific to *Thaumarchaeota* (E-EV_THAUM_) as well as of EV effects affecting all prokaryotes (E-EV_PROK_) (N_THAUM_ = *f*[E-EV_THAUM_, E-EV_PROK_], where EV_THAUM_ ⊂ E-EV_PROK_). If E-EV_PROK_ is considerably larger than E-EV_THAUM_, N_THAUM_ could depend largely upon E-EV_PROK_, which could potentially lead us to misinterpret the results. In such cases, EVs substantially affecting all prokaryotes might be erroneously identified as EVs that affect *Thaumarchaeota* specifically. Therefore, N_THAUM_ values normalized by the total 16S rRNA gene copy number (N_PROK_), R_THAUM_ = (N_THAUM_/N_PROK_), could provide better abundance estimates for an inference on E-EV_THAUM_ (R_THAUM_ ≈ *f*(E-EV_THAUM_). Correspondingly, when archaeal and bacterial *amoA* copy numbers (N_ARCH-amoA_ and N_BACT-amoA_) are compared, the raw values of archaeal *amoA* copy numbers (N_ARCH-amoA_) represent the effects of EVs upon both ammonia oxidizers generally (E-EV_PROK-amoA_) and archaeal ammonia oxidizers specifically (E-EV_ARCH-amoA_). Hence, only the relative proportion, R_ARCH-amoA_ = N_ARCH-amoA_/(N_ARCH-amoA_ + N_BACT-amoA_), could reflect the EV effects specifically upon archaeal ammonia oxidizers compared to those upon total ammonia oxidizers (R_ARCH-amoA_ ≈ *f*[E-EV_ARCH-amoA_]). As expected based upon the above considerations, the EV effects upon *Bacteria* (E-EV_BACT_) could not be distinguished from the EV effects upon total prokaryotes (E-EV_PROK_) in this study, because *Bacteria* represented a great majority of the prokaryotes in our soil samples and the sample variation in N_BACT_ could dominate the sample variation in N_PROK_ (if N_BACT_ → N_PROK_ [R_BACT_ ≈ 1], then E-EV_BACT_ ≈ E-EV_PROK_). Also, the sample deviation coefficient (RSD) of R_BACT_ was about 1/50 times those of R_ARCH_ and R_THAUM_, indicating that EV effects upon many bacterial phyla or a number of subphylum-level taxa (E-EV_*i*_) might be averaged or mutually cancelled out (∑[E-EV_*i*_] ≈ 0), which could explain the low RDA biplot score of R_BACT_. Similarly, because almost 50% of archaeal members in our soil samples were estimated to be *Thaumarchaeota*, N_THAUM_ became a major term in R_ARCH_. Hence, the responses of soil *Archaea* to EVs tended to resemble those of *Thaumarchaeota* in this study.

### Individual causal effect of environmental variables

The RDA inference of the potential causal relationships between the EVs and the RVs was further examined by Pearson’s correlation (symmetric analysis for reciprocal [bidirectional] relationships) and regression analyses (asymmetric analysis for causal [unidirectional] relationships). R_THAUM_ showed significant (*p* < 0.05) negative correlations to TC (*r* = −0.563), TN (*r* = −0.708), and TS (*r* = −0.612), and to WC (*r* = −0.599) (Table 2). Correlations between R_THAUM_ and EVs of inorganic nitrogen (NH_4_^+^-N and NO_3_^−^-N) were also significant (*p* < 0.05) and negative (−0.675 and −0.569, respectively). R_ARCH_ showed correlation results similar to those of R_THAUM_, but its correlation with NO_3_^−^-N was insignificant (*p* = 0.308). On the other hand, R_BACT_ showed significant (*p* < 0.05) positive correlations only with TN (*r* = +0.420) and NH_4_^+^-N (*r* = +0.474). The relationship between R_BACT_ and TN was not apparent in the RDA biplot, but was significant according to the symmetric analysis that did not assume an underlying causal relationship between these two variables. Moreover, R_BACT_ showed significant negative correlations with R_THAUM_ (*r* = −0.414, *p* < 0.05) and R_ARCH_ (*r* = −0.944, *p* < 0.05), while R_THAUM_ and R_ARCH_ had a significant positive correlation (*r* = +0.459, *p* < 0.05). In asymmetric analyses using simple linear regression, results similar to those of the correlation analyses were obtained. All the EVs that showed strong correlations with RVs also resulted in significant (*p* < 0.05, ANOVA) regression coefficients (slope, β_1_) (Table 3 and S2–S4 Figs). Each of these EVs explained >30% (R^2^ = 0.317–0.501) of the variation in R_THAUM_. WC appeared to have the most prominent effect (the largest slope, β_1_) on R_THAUM_ in nonstandardized scale, while TN was the strongest determinant of R_THAUM_ in the standardized (z-score–transformed) scale. Significant (*p* < 0.05, ANOVA) dependencies of R_ARCH_ on TC, TN, WC, and NH_4_^+^-N were also observed, but the explanatory powers of these EVs were relatively low (<30%, R^2^ = 0.170–0.302). Correlation results showed that R_BACT_ was affected only by TN and one of its inorganic forms, NH_4_^+^-N.

**Table 2:** Correlations between environmental variables and relative abundances of *Thaumarchaeota*, *Archaea*, and *Bacteria*. Lower left half, Pearson correlation coefficients (*r*); upper right half, *p* values.

	R_THAUM_	R_ARCH_	R_BACT_	Temp	pH	WC	TC	TN	NH_4_^+^-N	NO_3_^−^-N	TP	TS
*Thaumarchaeota*(R_THAUM_)												
*Archaea*(R_ARCH_)	**0.459** ^a^											
*Bacteria*(R_BACT_)	**-0.414**	**-0.944**										
Temperature (Temp)	-0.022	-0.275	0.352									
pH	-0.141	0.176	-0.136	-0.057								
Water content (WC)	**-0.599**	**-0.413**	0.317	0.022	0.007							
Total carbon (TC)	**-0.563**	**-0.396**	0.315	0.045	0.071	**0.640**						
Total nitrogen (TN)	**-0.708**	**-0.502**	**0.420**	0.016	0.247	**0.834**	**0.653**					
Ammonium-nitrogen (NH_4_^+^-N)	**-0.675**	**-0.550**	**0.474**	0.102	0.074	**0.879**	**0.691**	**0.946**				
Nitrate-nitrogen (NO_3_^−^-N)	**-0.569**	-0.204	0.082	**-0.418**	0.292	**0.583**	**0.523**	**0.701**	**0.651**			
Total phosphorus (TP)	-0.110	-0.240	0.216	0.045	0.020	0.230	0.254	**0.397**	**0.481**	0.332		
Total sulfur (TS)	**-0.612**	-0.297	0.197	-0.065	0.346	**0.728**	**0.481**	**0.820**	**0.764**	**0.744**	0.304	

^a^ Significant (*p* < 0.05) correlations are displayed in bold.

**Table 3:** Results of regressions between environmental variables and relative abundances of *Thaumarchaeota*, *Archaea*, and *Bacteria*.

Variables		Regression coefficient (β_1_)	Standardized Regression	Coefficient of determination (R^2^)	Analysis of variance (ANOVA)	
Response (dependent)	Explanatory (independent)				*F* statistic	*P* value

			coefficient			
*Thaumarchaeota* (R_THAUM_)	Water content	**-1.081** ^a^	**-0.599**	**0.359**	**14.0**	**0.001**
	Total carbon	**-0.504**	**-0.563**	**0.317**	**11.6**	**0.002**
	Total nitrogen	**-0.519**	**-0.708**	**0.501**	**25.1**	**<0.001**
	NH_4_^+^-N	**-0.517**	**-0.675**	**0.456**	**20.9**	**<0.001**
	NO_3_^−^-N	**-0.759**	**-0.569**	**0.324**	**12.0**	**0.002**
	Total sulfur	**-0.584**	**-0.612**	**0.374**	**15.0**	**0.001**
*Archaea* (R_ARCH_)	Water content	**-0.467**	**-0.413**	**0.170**	**5.1**	**0.032**
	Total carbon	**-0.222**	**-0.396**	**0.157**	**4.6**	**0.041**
	Total nitrogen	**-0.231**	**-0.502**	**0.252**	**8.4**	**0.008**
	NH_4_^+^-N	**-0.264**	**-0.550**	**0.302**	**10.8**	**0.003**
	NO_3_^−^-N	-0.170	-0.204	0.041	1.1	0.308
	Total sulfur	-0.178	-0.297	0.088	2.4	0.132
*Bacteria* (R_BACT_)	Water content	0.010	0.317	0.100	2.8	0.107
	Total carbon	0.005	0.315	0.099	2.8	0.109
	Total nitrogen	**0.005**	**0.420**	**0.176**	**5.4**	**0.029**
	NH_4_^+^-N	**0.006**	**0.474**	**0.225**	**7.3**	**0.012**
	NO_3_^−^-N	0.002	0.082	0.007	0.2	0.685
	Total sulfur	0.003	0.197	0.039	1.0	0.326

^a^ Significant (*p* < 0.05) correlations are displayed in bold.

Although pH was previously suggested as an important factor that could shape the niche of *Thaumarchaeota* (or AOA) [21, 24, 58–63], no evidence suggesting a role of pH in controlling R_THAUM_ was observed in this study. It was considered that the insignificant relationship observed between pH and R_THAUM_ could arise from the relatively small sample variation (as reflected by RSD) of pH in our soil samples compared to those of other EVs that showed prominent effects on R_THAUM_. The lack of a significant relationship between temperature and R_THAUM_ might also be attributed to our limited sampling; our soil samples were collected from temperate sites in Korea. On the other hand, despite the fact that the sample variation of TP was comparable to those of the prominent EVs (TC, TN, TS, and WC), TP appeared not to affect R_THAUM_. Although the causal effects of pH, temperature, and TP on R_THAUM_ appeared insignificant in this study, the roles of these EVs in shaping the niche of soil *Thaumarchaeota* should be further examined by comprehensive surveys using wider ranges of sample EVs than those used herein.

To the best of our knowledge, all the physiologically described members of *Thaumarchaeota* were cultured (or enriched) in growth media containing bicarbonate (hydrogen carbonate, HCO_3_^−^) as the sole source of carbon [3, 6–16], suggesting an autotrophic lifestyle of *Thaumarchaeota*. Evidence of autotrophy by *Thaumarchaeota* include the genomic components of a modified 3-hydroxypropionate/4-hydroxybutyrate (3HP/4HB) pathway [6, 8, 12, 16, 64–66] and a reductive (or reverse) tricarboxylic acid (rTCA) cycle [66–68] found in genomes of the cultured (or enriched) members of *Thaumarchaeota*. Along with this genomic evidence, experimental results obtained using stable isotope probing confirmed the autotrophic growth and activity of *Thaumarchaeota* [27, 69]. However, heterotrophy (mixotrophy, more likely) has been also suggested for thaumarchaeotal physiology. In addition to genes encoding the 3HP/4HB pathway, oxidative TCA cycle genes were retrieved in genomes of *C*. *symbiosum* [64, 67] and *N*. *maritimus* [66]. Moreover, many physiological studies suggested that *Thaumarchaeota* has the potential to uptake OM for growth [16, 57, 70–74]. However, regardless of whether *Thaumarchaeota* is autotrophic or mixotrophic, cultures of *Thaumarchaeota* have been shown to be susceptible to organic carbon content in culture media, and our result regarding the effect of TC on R_THAUM_ is consistent with these findings. Namely, addition of organic compounds, even in very low concentrations, inhibited the growth of *N*. *maritimus* [3], and slowed the ammonia oxidation of *N*. *yellowstonii* [7]. Although Tourna *et al*. [16] reported that growth of *N*. *viennensis* was enhanced by the addition of a small amount of pyruvate to the culture medium, such growth-stimulating effects were observed only at very low concentrations (~0.05 mM). Suppressive effects of organic carbon on the abundance of *Thaumarchaeota* were also observed in a study by Wessen *et al*. [75]. They reported a significant (*p* < 0.05) negative correlation (*r* = −0.60) between *Thaumarchaeota* (AOA) abundance and organic carbon content in soil samples; our results are consistent with this finding. Di *et al*. [76] also reported that the populations of *Thaumarchaeota* (AOA) were more numerous in soils with lower organic carbon content. In addition to such growth-suppressing effects, organic carbon content has been shown to be negatively related to *Thaumarchaeota* (AOA) species richness [69].

Similar to the effect of TC on *Thaumarchaeota*, TN as well as the two inorganic forms of nitrogen (NH_4_^+^-N, and NO_3_^−^-N) negatively affected R_THAUM_ in this study. While Hofferle *et al*. [22] and Stopnisek *et al*. [77] reported that ammonium concentration had a marginal or nonexistent effect on thaumarchaeotal (AOA) abundance in soil, a strong negative relationship between thaumarchaeotal growth and ammonium concentration in soil has been observed in many environmental studies [20, 69, 78], indicating that *Thaumarchaeota* prefer low ammonia concentration for growth and ammonia oxidation activity. Many studies on cultured members of *Thaumarchaeota* also showed that thaumarchaeotal growth rate decreased with increasing ammonium concentration. Growth of *N*. *gargensis* and of *N*. *maritimus* was inhibited at very low ammonium concentrations (2 mM and 3 mM, respectively) [8, 79]. Studies by Tourna *et al*. [16], Jung *et al*. [9], and Morley *et al*. [12] demonstrated growth inhibition of *Thaumarchaeota* (*N*. *viennensis*, *N*. *koreensis*, and *N*. *devanaterra*) at ammonium concentrations that were slightly higher (>20–50 mM), but still much lower than those that inhibited AOB growth. Some AOB were reported to be able to grow at >200 mM NH_4_^+^ [80–82]. Moreover, Tourna *et al*. [16] considered that the accumulation of toxic metabolic intermediates including nitrate (NO_2_^−^) might inhibit the growth of AOA (*N*. *viennensis*) at high (>3.5 mM) ammonium concentrations. On the other hand, unlike those of ammonium, the effects of nitrate and TN upon thaumarchaeotal growth in culture media or upon estimates of its abundance in the environment have not yet been well studied. This could possibly be due to the fact that most physiological and ecological studies concerning the effects of nitrogen upon *Thaumarchaeota* have been focused mainly on ammonium (or ammonia), the substrate of ammonia oxidation. In our study, although we did not determine the ammonia oxidation rate, the NO_3_^−^/NH_4_^+^ ratio, which might partly explain the rate of nitrification, correlated positively to R_THAUM_ (*r* = 0.456, *p* = 0.017) and negatively to organic carbon content (*r* = −0.512, *p* = 0.006).

### Indirect but ultimate causal effect of OM

Considering that most soil nitrogen is in organic form [83], the effect of TN on R_THAUM_ could overlap with the effect of TC on R_THAUM_. Because no samples in this study were collected from sites to which fertilizer (artificial sources of nitrogen and carbon) had been applied, both the TN and TC in our samples were thought to have originated from indigenous OM such as plant debris and other types of indigenous biomass, under the assumption that carbon fixation and nitrogen fixation are the only routes of carbon and nitrogen input to our soil samples. Thus, the level of OM could determine the levels of TC and TN, and the levels of TC and TN could subsequently be interrelated. In fact, TC and TN showed a significant positive correlation (*r* = 0.653, *p* < 0.05) in this study. Bearing in mind these points, we questioned if the observed EV effects of TC or TN on R_THAUM_ were indirect causal effects. Even though only TN actually affects *Thaumarchaeota* and TC actually does not, a superficial relationship between TC and R_THAUM_ likely appeared because TC correlated with TN. Hence, we applied path analysis [54, 55] to determine the direct and indirect causal effects of TC and TN upon R_THAUM_, as well as to test their causal ordering.

**Fig 2:**
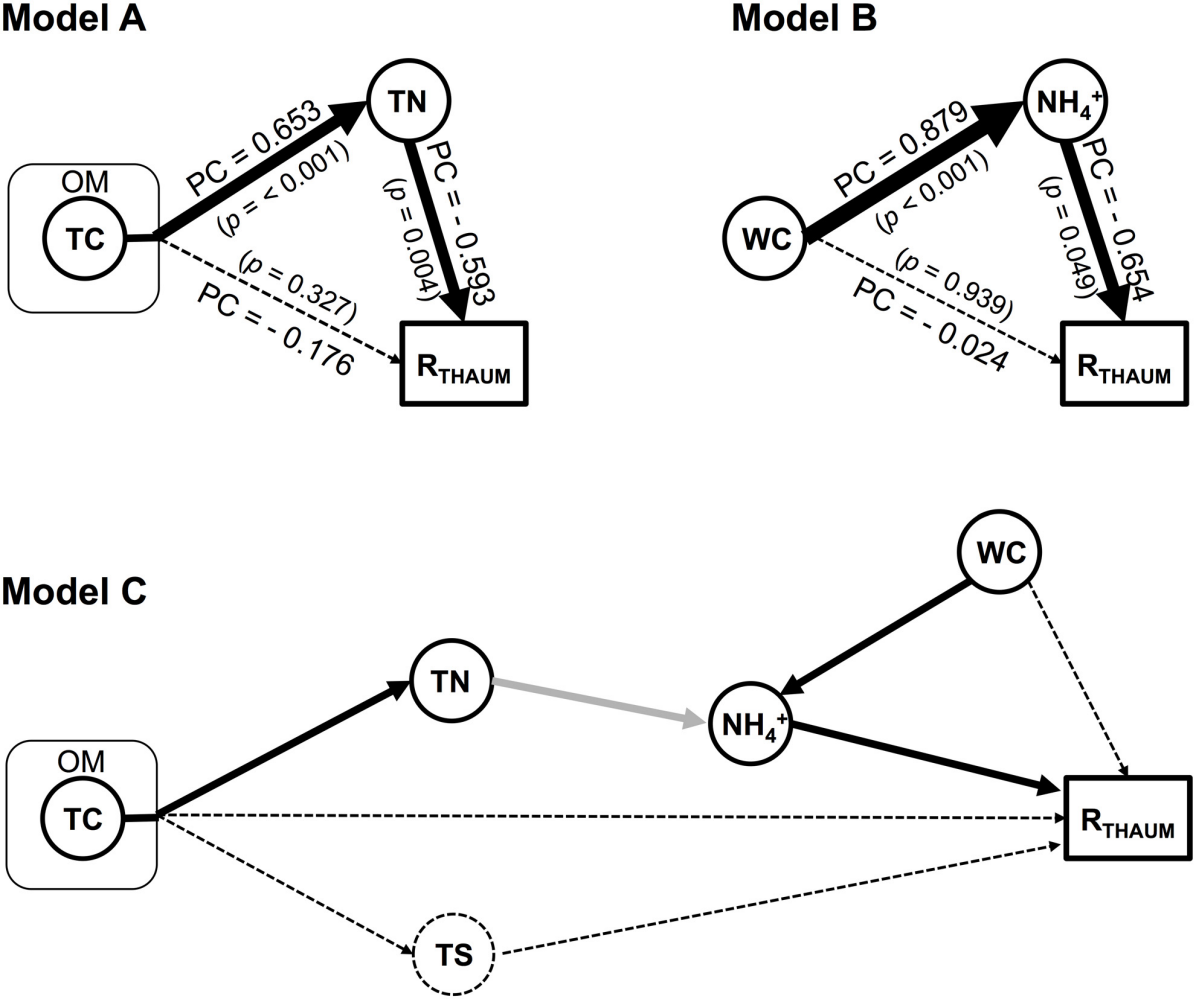
Path diagram of the effects of environmental variables upon the relative abundance of *Thaumarchaeota* in soil. Path model A shows the causal effects of TC and TN upon R_THAUM_, and path model B shows the causal effects of WC and NH_4_^+^-N on R_THAUM_. Path model C combines A and B in a single concatenated model; the link between models A and B is indicated by a grey arrow. Causal ordering is represented by arrows. Solid lines and dashed lines respectively indicate direct and indirect causal effects in path models A and B, and the thickness of each solid line represents its PC. In path model C, all environmental variables are included that showed significant effects on R_THAUM_ in simple linear regression analysis.

In the path analysis, we found a significant (χ^2^_GoF_ < 0.001, RMSEA < 0.001) path model that explained the causal effects of TC and TN upon R_THAUM_ (Fig 2). In this path model, only TN directly affected R_THAUM_ (PC = −0.593, *p* = 0.004). The effect of TC upon R_THAUM_ was indirect (PC = −0.176, *p* = 0.356), and TN was a mediator EV. This result suggested that the relationship between TC and R_THAUM_ was chiefly an indirect effect mediated by TN. In addition, when bivariate covariations among the variables were decomposed (Table 4), only 31.3% of the total covariation between TC and R_THAUM_ was explained by direct causation, whereas 83.8% of the total covariation between TN and R_THAUM_ was explained by direct causation. Note that a finding of an indirect effect does not mean that there is no effect, but does mean that the effect is mediated by another variable. Additionally, we supposed that the TC level measured in this study (recall that TC ≈ TOC, as total inorganic carbon was below the detection limit for all samples) could be used as a proxy for the level of OM in our soil samples. Since carbon content is generally about 10 times that of nitrogen content in natural OM (i.e., its C/N ratio is ca. 10), the observed TC effect was considered to dominate the expected OM effect. Besides, the TC level strongly corresponded to the OM level in our preliminary study. Thus, in terms of causal ordering, we concluded that OM might be an indirect but ultimate EV controlling the abundance of *Thaumarchaeota*, and that this causal effect of OM could be mediated by TN. Although insignificant and weak, the direct causal effect of OM upon R_THAUM_ might correspond to the growth-suppressive effect of organic carbons (mostly carbohydrates) observed in the culture-based studies mentioned above. On the other hand, in the path analysis that was applied to TS under the assumption that TS also originates from OM, no significant path models were found. This result suggested that our assumption regarding TS could be false, or that there might be latent mediator EVs that were not measured in this study (e.g., SO_4_^−^ and HS^−^). A recent study by Park *et al*. [65] reported that AOA were selectively enriched over AOB in the presence of sulfur-oxidizing bacteria. Although they concluded that the selective enrichment of AOA was likely due to oxygen depletion caused by the rapid growth of sulfur-oxidizing bacteria, an oxidized sulfur species might enhance the growth of AOA, or, inversely, a reduced sulfur species might inhibit the growth of AOA.

**Table 4:** Direct and indirect causal effects of environmental variables upon the relative abundance of *Thaumarchaeota* in hypothesized path models.

Path Model	Causal direction^a^	Total covariation	Causal covariation (effect)			Noncausal covariation
			Direct	Indirect	Total	
A	TC → R_THAUM_	-0.563	-0.176	-0.387	-0.563	0.000
	TN → R_THAUM_	-0.708	**-0.593** ^b^	0.000	-0.593	-0.115
	TC → TN	0.653	**0.653**	0.000	0.653	0.000
B	WC → R_THAUM_	-0.599	-0.024	-0.575	-0.599	0.000
	NH_4_^+^ → R_THAUM_	-0.675	**-0.654**	0.000	-0.654	0.021
	WC → NH_4_^+^	0.879	**0.879**	0.000	0.879	0.000

^a^ Causal ordering is represented by arrows.

^b^ Significant (*p* < 0.05, t-statistic calculated from MLR using the OLS method) direct effects are displayed in bold.

Except for the nutrient-related EVs, only WC showed a significant correlation with R_THAUM_ (*r* = −0.599, *p* < 0.05) and was a significant EV explaining the variance of R_THAUM_ (R^2^ = 0.359, *p* < 0.05), implying that the members of *Thaumarchaeota* in soil could prefer low-WC conditions or that they could be less vulnerable to desiccation stress than their competitors. Microorganisms exposed to low-WC environments must possess mechanisms to avoid water loss by osmosis, and a well-known strategy to maintain turgor is to accumulate intracellular compatible solutes (osmolytes). All xerophiles (including some halophiles) produce and accumulate low-molecular-mass organic compounds that have osmotic potential (organic osmolytes) [84, 85]. In *Thaumarchaeota*, genes coding for the biosynthesis of ectoine, an organic osmolyte, were observed in the genome of *N*. *maritimus* [66]. However, no experimental evidence has been reported regarding xerophilic (or xerotolerant) characteristics of *Thaumarchaeota*. Even though not being xerophiles, soil microorganisms are generally considered to be adapted to cope with water stress because water levels in soil fluctuate. Hence, we first hypothesized that the terrestrial members of *Thaumarchaeota* might have better mechanisms to respond to desiccation stress than their competitors do. However, this hypothesis was inconsistent with our conclusions regarding TC and TN because WC showed positive correlations with TC and TN, both of which negatively affect R_THAUM_. To avoid this contradiction, an alternative hypothesis on the effect of WC on R_THAUM_ was formulated by using path analysis.

In the path analyses employing WC as a fixed explanatory variable and the other EVs as in-and-out variables, we found a significant (χ^2^_GoF_ < 0.001, RMSEA < 0.001) path suggesting an effect of WC, mediated by ammonium, upon R_THAUM_ (Fig 2). Unlike the SLR model, in which WC appeared to be one of the significant determinants of R_THAUM_, the direct effect of WC on R_THAUM_ in this path was almost zero (PC = 0.024) and insignificant (*p* = 0.939). WC directly affected ammonium concentration (PC = 0.879, *p* < 0.001) rather than R_THAUM_, and the ammonium concentration subsequently affected R_THAUM_. Only 4.0% of the total covariation between WC and R_THAUM_ was explained by direct causation, whereas 96.9% of the total covariation between ammonium and R_THAUM_ was explained by direct causation. We hypothesized based on the path analysis results that WC negatively affected R_THAUM_ by means of the mineralization of organic nitrogen (ammonification). Considering that WC positively regulates the rate of nitrogen mineralization (a major route of inorganic nitrogen input in soil) [86–89], low WC could result in increased R_THAUM_ by decreasing the ammonium level, because *Thaumarchaeota* might prefer low-ammonium conditions. This hypothesis was consistent with our suggestion that, among the nutrient-related EVs, nitrogen content might be the most direct factor controlling *Thaumarchaeota* in soil. To present a possible scenario describing all the prominent relationships between soil EVs and *Thaumarchaeota* identified in this study, we developed a combined path model that can be examined in future studies (Fig 2). Although this model was not significant (χ^2^_GoF_ = 60.9, RMSEA = 0.377), we tried to incorporate all the direct and indirect causal effects of EVs on R_THAUM_ in this single model. There might be latent variables (e.g., inorganic sulfur/phosphorus) that were not included in this study, or missing links between variables.

## Concluding Remarks

The relative abundance patterns of *Thaumarchaeota* in this study demonstrated the oligotrophic lifestyle of *Thaumarchaeota* in soil. The oligotrophy of *Thaumarchaeota* might be attributed to their enzymes and transporters with high affinity, which provide them a competitive advantage under nutrient-limiting conditions. In fact, it has been suggested that the reason why *Thaumarchaeota* favor low ammonium concentrations is their exceptionally low half-saturation constant (*K*_*m*_ = 0.13–0.61 μM) of ammonia monooxygenase [9, 65, 79], which was 1/40 to 1/10,000 times that of the *K*_*m*_ values of AOB (*K*_*m*_ = 30–1,300 μM) [65, 90, 91]. Another hypothesis regarding energy metabolism was also previously formulated based on thaumarchaeotal genomic sequences to explain the ammonium oligotrophy of *Thaumarchaeota*. Genomes of *Thaumarchaeota* (*C*. *symbiosum* and *N*. *maritimus*) [64, 66] were reported to lack genes encoding the hydroxylamine–ubiquinone redox module, which has been considered indispensable in electron recycling during the initial oxidation of ammonium, generating proton motive force and reducing power. To explain this unusual genomic feature, Walker *et al*. [66] hypothesized that *Thaumarchaeota* might not require the electron recycling module because archaeal ammonia monooxygenase produces nitroxyl hydride (HNO) instead of hydroxylamine (NH_2_OH), suggesting that this simplified ammonia oxidation process could provide an ecological advantage to *Thaumarchaeota* under ammonium-limited conditions. Along with its high affinity to substrate and its efficient energy metabolism, *Thaumarchaeota* might have other characteristics common in oligotrophs (e.g., high vulnerability to toxic metabolites rapidly accumulated under nutrient-rich conditions and to energy depletion caused when excessive transportable nonmetabolic substances suddenly become available) [92], a topic that remains to be studied.

Although published thaumarchaeotal genome sequences have shown only one copy of the 16S rRNA gene in each thaumarchaeotal genome [6, 10, 11, 13, 14, 64, 66, 93], many other prokaryotes have been reported to possess multiple copies of the 16S rRNA gene in each cell [94]. Errors related to such variations in the 16S rRNA gene copy number per genome among prokaryotic taxa could affect our results on the relative abundance of *Thaumarchaeota*, possibly causing underestimation of the actual abundance of *Thaumarchaeota* in soil. Also, environmental variables measured in this study do not represent the full spectrum of environmental variables that *Thaumarchaeota* interact with. Hence, our conclusion might be only a snapshot of the ecology of *Thaumarchaeota*. Comprehensive ecological studies overcoming our limitations, and future research on the physiology and molecular biology of cultured/enriched cells, will allow us to more fully understand the ecological niche of the intriguing phylum *Thaumarchaeota*.

## Supporting Information

S1 Fig Standard curves of C_T_ values versus (A) thaumarchaeotal, (B) archaeal, and (C) bacterial 16S rRNA gene copy numbers. Solid lines indicate regression curves and error bars indicate standard deviations obtained in quadruplicate experiments. The R^2^ values of regression curves were 0.999 for *Thaumarchaeota*, 0.994 for *Archaea*, and 0.998 for *Bacteria*, respectively.
(PDF)

S2 Fig Significant (*p* < 0.05, ANOVA) regression curves indicating the effects of WC, TC, TN, NH_4_^+^-N, NO_3_^−^-N, and TS upon R_THAUM_. (PDF)

S3 Fig Significant (*p* < 0.05, ANOVA) regression curves indicating the effects of WC, TC, TN, and NH_4_^+^-N upon R_ARCH_. (PDF)

S4 Fig Significant (*p* < 0.05, ANOVA) regression curves indicating the effects of TN and NH_4_^+^-N upon R_BACT_. (PDF)

S1 Table *In silico* evaluation (percent matched 16S rRNA gene sequences)^a^ of primer pairs, used to quantify the abundance of *Thaumarchaeota*. (PDF)

S2 Table *In silico* evaluation (percent matched 16S rRNA gene sequences)^c^ of universal primers, used to quantify the abundance of *Archaea* and *Bacteria*. (PDF)
