# Supplementary material for: Environmental Variables Shaping the Ecological Niche of Thaumarchaeota in Soil: Direct and Indirect Causal Effects
Source: PLoS One. 2015 Aug 4;10(8):e0133763. doi: 10.1371/journal.pone.0133763 (PMC4524719; doi:10.1371/journal.pone.0133763)
Supplement: S1 Fig — Solid lines indicate regression curves and error bars indicate standard deviations obtained in quadruplicate experiments. The R2 values of regression curves were 0.999 for Thaumarchaeota, 0.994 for Archaea, and 0.998 for Bacteria, respectively. (PDF) [file pone.0133763.s001.pdf]

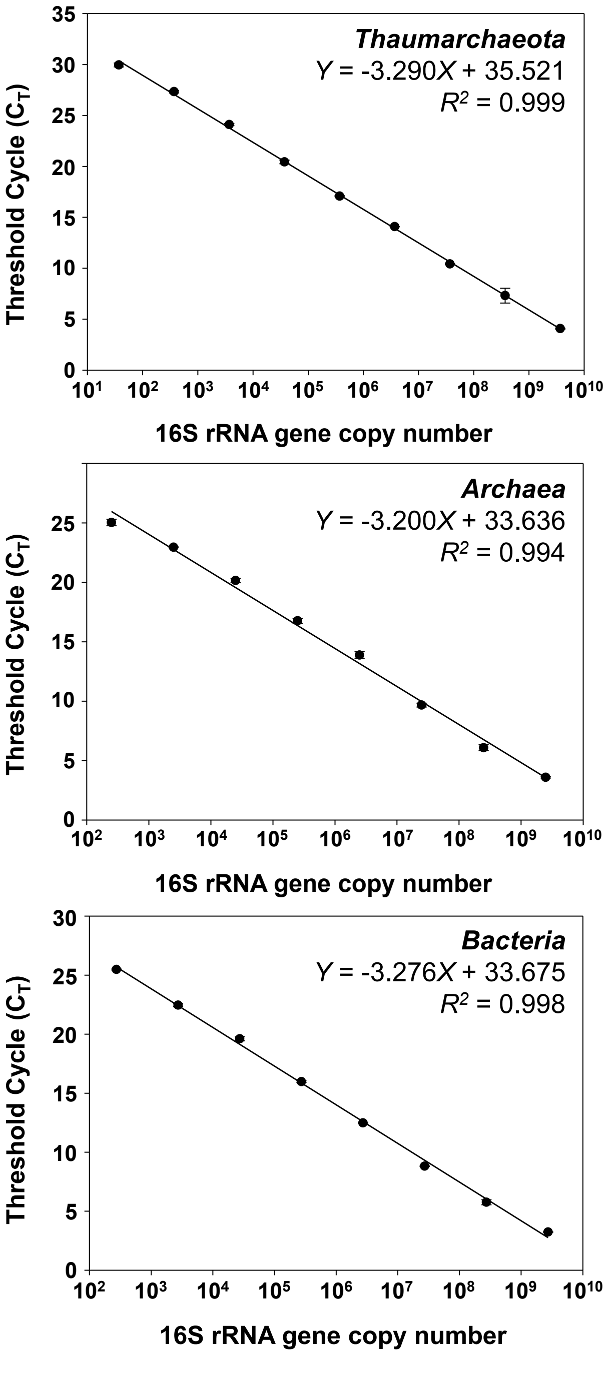

S1 Fig. Standard curves of C_T values versus (A) thaumarchaeotal, (B) archaeal, and (C) bacterial 16S rRNA gene copy numbers. Solid lines indicate regression curves and error bars indicate standard deviations obtained in quadruplicate experiments. The R^2 values of regression curves were 0.999 for *Thaumarchaeota*, 0.994 for *Archaea*, and 0.998 for *Bacteria*, respectively.
