# Supplementary material for: Environmental Variables Shaping the Ecological Niche of Thaumarchaeota in Soil: Direct and Indirect Causal Effects
Source: PLoS One. 2015 Aug 4;10(8):e0133763. doi: 10.1371/journal.pone.0133763 (PMC4524719; doi:10.1371/journal.pone.0133763)
Supplement: S2 Fig — (PDF) [file pone.0133763.s002.pdf]

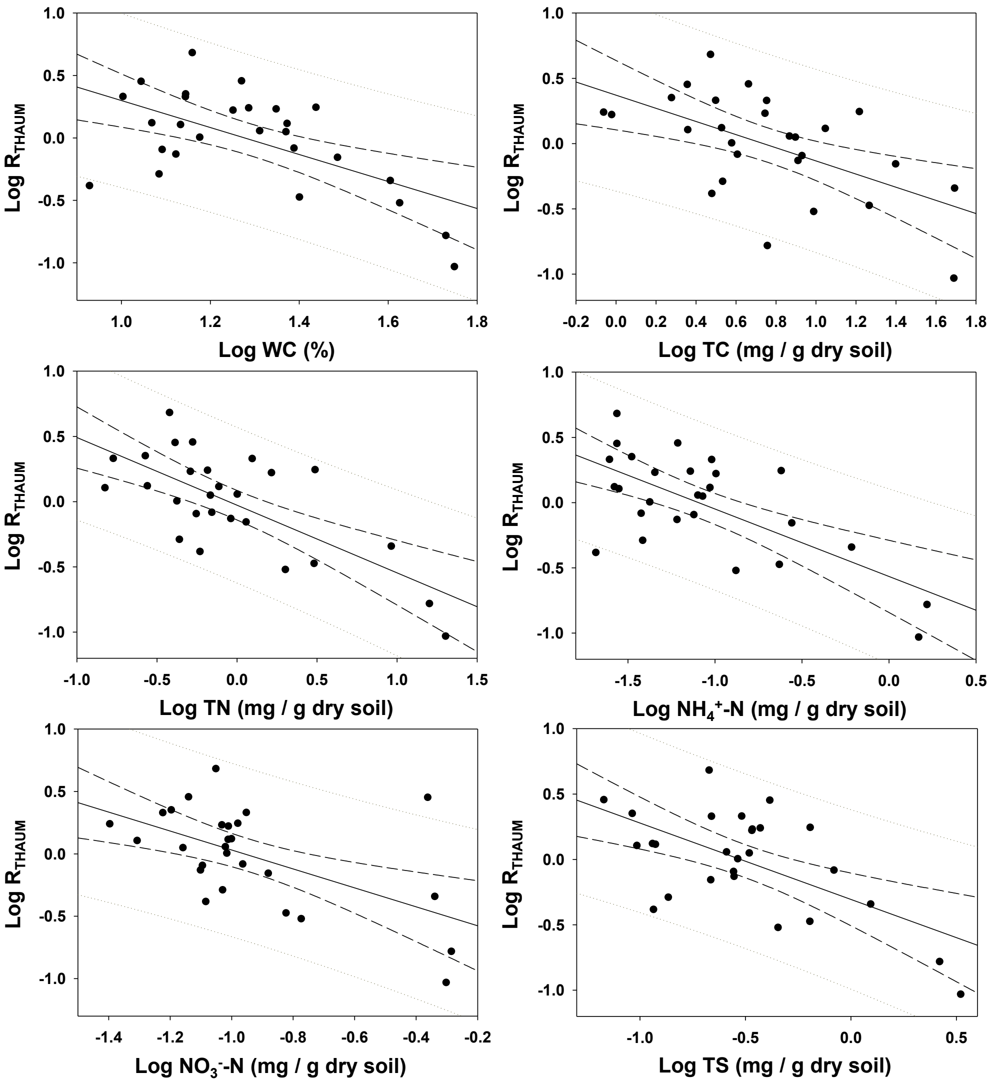

S2 Fig. Significant ($p < 0.05$, ANOVA) regression curves indicating the effects of WC, TC, TN, NH_4^+-N, NO_3^--N, and TS upon R_{THAUM}.
