# Supplementary material for: Environmental Variables Shaping the Ecological Niche of Thaumarchaeota in Soil: Direct and Indirect Causal Effects
Source: PLoS One. 2015 Aug 4;10(8):e0133763. doi: 10.1371/journal.pone.0133763 (PMC4524719; doi:10.1371/journal.pone.0133763)
Supplement: S1 Table — (PDF) [file pone.0133763.s005.pdf]

S1 Table. *In silico* evaluation (percent matched 16S rRNA gene sequences)[a] of primer pairs, used to quantify the abundance of *Thaumarchaeota*.

Taxa	No. of sequences used for evaluation	PCR primer pair used in this study (THAUM-494-ARC917R)[b]			Previously used PCR primer pairs for quantifying *Thaumarchaeota*					
					Primer pair 771F-957R			Primer pair MCGI391f-MCGI554r		
		Primer pair	THAUM-494[c]	ARC917R[d]	Primer pair	771F[e]	957R[e]	Primer pair	MCGI391f[f] (MGI391)	MCGI554r[f] (Cren537)
Crenarchaeota	872	0.9	0.9	76.3	1.0	10.9	0.6			
Euryarchaeota	6,072			91.8	$\approx$0		$\approx$0			
Korarchaeota	88									
Nanoarchaeota	3									
Thaumarchaeota[g]	1,549	**89.3**	92.9[a]	96.1	21.9	94.6	22.4	33.7	35.8	65.5
FSCG	17			100		76.5				
HWCG-III	25	32.0	36.0	92.0	76.0	100				
MG-I	1,100	**92.9**	96.6	96.0	0.2	95.2	0.1	47.5	50.4	92.3
RC	7			100	57.1	85.7				
SAGMCG-I	40	**97.5**	100	97.5	7.5	87.5	10.0			
SCG	355	**87.6**	90.7	96.3	87.3	94.1	96.1			
UT-I	2	50.0	100	50.0	100.0	100	50.0			
UT-II	1	**100.0**	100	100		100				
UT-III	2	**100.0**	100	100		100				
DSAG[h]	326			93.3						
THSCG[h]	50			68.0						
MCG[h]	483			93.2		28.0	0.2			
UG[h]	12			91.7						
Unclassified *Archaea*	272			78.3	36.4	41.2	0.4			
Domain *Bacteria*	667,899									

[a] Estimated by Hong *et al*. (Hong *et al*., 2014)

[b] Forward primer, THAUM-494; reverse primer, ARC917R

[c] *Thaumarchaeota*-directed primer (3′-GAATAAGGGGTGGGCAAGT-5′, Hong *et al*., 2014)

[d] Archaeal universal primer (3′-GAATTGGCGGGGGAGCAC-5′, Loy *et al*., 2002)

[e] Developed by Ochsenreiter *et al*. (Ochsenreiter *et al*., 2003)

[f] Developed by Auguet *et al*. (Auguet *et al*., 2012)

[g] Thaumarchaeotal subgroup sequences classified by Hong *et al*. (Hong *et al*., 2014). FSCG, Forest Soil Crenarchaeotic Group; HWCG-III, Hot Water Crenarchaeotic Group III; MG-I, Marine Group I; RC, Rice Cluster; SAGMCG-I, South African Gold Mine Crenarchaeotic Group I; SCG, Soil Crenarchaeotic Group; UT-I, -II, and -III, Unclassified Thaumarchaeota-I, -II, and -III.

[h] Environmental archaeal groups classified by Hong *et al*. (Hong *et al*. 2014). DSAG, Deep Sea Archaeal Group; THSCG, Terrestrial Hot Spring Crenarchaeotic Group; MCG, Miscellaneous Crenarchaeotic Group; UG, Unclassified Group.
