# Supplementary material for: Environmental Variables Shaping the Ecological Niche of Thaumarchaeota in Soil: Direct and Indirect Causal Effects
Source: PLoS One. 2015 Aug 4;10(8):e0133763. doi: 10.1371/journal.pone.0133763 (PMC4524719; doi:10.1371/journal.pone.0133763)
Supplement: S2 Table — (PDF) [file pone.0133763.s006.pdf]

S2 Table. *In silico* evaluation (percent matched 16S rRNA gene sequences)[c] of universal primers, used to quantify the abundance of *Archaea* and *Bacteria*.

Taxa	No. of sequences used for evaluation	Archaeal universal primers pair[a]	Bacterial universal primers pair[b]
		ARC806-ARC915	Eub338-BAC515
Archaea	25,760	86.6[c]	0.0
Crenarchaeota	2,202	72.8	0.0
Euryarchaeota	14,339	87.6	0.0
Korarchaeota	92	0.0	0.0
Nanoarchaeota	139	0.0	0.0
Thaumarchaeota	7,761	95.7	0.0
Unclassified *Archaea*	1,200	58.2	0.0
Bacteria	1,408,156	0.0	76.3
Actinobacteria	212,832	0.0	62.9
Aquificae	968	0.0	4.6
Bacteroidetes	173,039	0.0	84.9
Caldiserica	218	0.0	0.5
Chlamydiae	317	0.0	0.0
Chlorobi	381	0.0	0.0
Chloroflexi	21,334	0.0	12.2
Chrysiogenetes	14	0.0	71.4
Deferribacteres	398	0.0	95.5
Deinococcus-Thermus	2,059	0.0	93.1
Dictyoglomi	19	0.0	0.0
Elusimicrobia	356	0.0	58.7
Fibrobacteres	823	0.0	89.2
Fusobacteria	1,181	0.0	924.6
Gemmatimonadetes	1,335	0.0	59.3
Lentisphaerae	1,792	0.0	0.2
Nitrospirae	1,647	0.0	58.5
Planctomycetes	11,190	0.0	0.9
Proteobacteria	398,053	0.0	80.6
Spirochaetes	10,229	0.0	17.1
Synergistetes	1,231	0.0	79.9
Tenericutes	4,632	0.0	3.1
Thermodesulfobacteria	106	0.0	88.7
Thermotogae	648	0.0	53.5
BRC1	395	0.0	13.7
Parcubacteria	150	0.0	0.0
Microgenomates	111	0.0	0.0
SR1	319	0.0	78.4
Candidatus Saccharibacteria	2,350	0.0	0.1
Latescibacteria	550	0.0	72.4
Armatimonadetes	1,112	0.0	2.6
Verrucomicrobia	10,137	0.0	0.1
Acidobacteria	15,398	0.0	66.9
Firmicutes	459,584	0.0	88.9
Cyanobacteria /Chloroplast	24,493	0.0	66.4
Marinimicrobia	982	0.0	71.2
Aminicenantes	1,464	0.0	87.2
Omnitrophica	19	0.0	10.5
Acetothermia	39	0.0	0.0
Poribacteria	103	0.0	3.9
Atribacteria	65	0.0	96.9
Cloacimonetes	167	0.0	9.6
Candidatus Calescamantes	3	0.0	66.7
WPS-1	769	0.0	0.0
WPS-2	97	0.0	0.0
Hydrogenedentes	446	0.0	2.0
ZB3	75	0.0	49.3
Ignavibacteriae	739	0.0	91.7
Nitrospinae	525	0.0	95.2
Unclassified *Bacteria*	32,285	0.0	36.2

[a] Archaeal universal primer pair: Forward primer, ARC806 (3′-GGACTACVSGGGTATCTAAT-5′, Casamayor *et al*., 2002); reverse primer, ARC915 (3′-GTGCTCCCCCGCCAATTCCT-5′, Takai and Horikoshi, 2000)

[b] Bacterial universal primer pair: Forward primer, Eub338 (3′-CTGCTGCCTCCCGTAGGAGT-5′, Nadkarni *et al*., 2002); reverse primer, BAC515 (3′-GTGCCAGCAGCCGCGGTAATACG-5′, Stahl and Amann, 1991)

[c] Estimation using RDP’s ProbeMatch software.
